# Case Report of Torsion of Cryptorchid Testis Causing Abdominal Pain

**DOI:** 10.5811/cpcem.2021.12.54845

**Published:** 2022-01-28

**Authors:** Hannah Newhouse, Joseph Minardi, Frederic Rawlins

**Affiliations:** * West Virginia University Camden Clark Medical Center, Department of Emergency Medicine, Parkersburg, West Virginia; † Virginia College of Osteopathic Medicine, Department of Emergency Medicine, Blacksburg, Virginia

**Keywords:** Cryptorchid, undescended, testicle, torsion, case report

## Abstract

**Introduction:**

A myriad of pathologies can cause abdominal pain. Genitourinary causes including testicular torsion must be considered.

**Case Report:**

In this report, we present a 17-year-old male evaluated in the emergency department for lower abdominal pain. After physical exam, computed tomography, and ultrasound were completed, torsion of undescended testicle within the inguinal canal was diagnosed. Surgical exploration revealed a twisted, ischemic testis, and subsequent orchiectomy was performed.

**Conclusion:**

This case highlights the importance of a thorough genitourinary exam in patients with lower abdominal pain.

## INTRODUCTION

Representing approximately 8% of all visits, abdominal pain is the most common complaint encountered in the emergency department (ED).^1^ While the etiology may vary, genitourinary sources of pain must be considered including testicular torsion. Although testicular torsion frequently presents as acute scrotal pain, torsion of an undescended testicle may present as non-specific lower abdominal or groin pain. We present a case of torsion of a cryptorchid testis in an adolescent male.

## CASE REPORT

A healthy 17-year-old male presented to the ED with left lower-quadrant abdominal pain. The patient experienced onset of severe pain upon awakening two days earlier with associated nausea and vomiting. The pain had persisted although vomiting had since resolved. He had a history of constipation but recently had normal bowel movements. He denied hematuria, dysuria, fever, or any other symptoms. Vital signs were normal. On physical exam, he had a firm, non-reducible mass in the left inguinal region with tenderness and guarding to the left lower quadrant. Testicular exam was not performed initially.

**Image 1:**
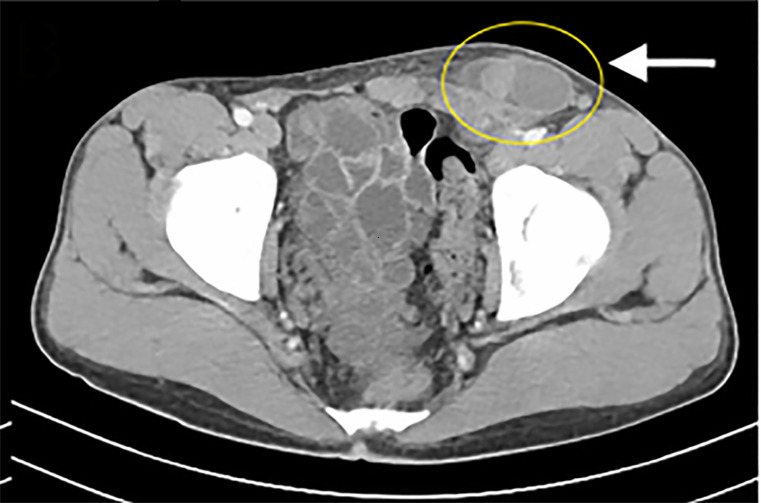
Axial computed tomography at the lower margin of the pelvis with the cryptorchid testicle shown in the left inguinal canal (circle).

**Image 2:**
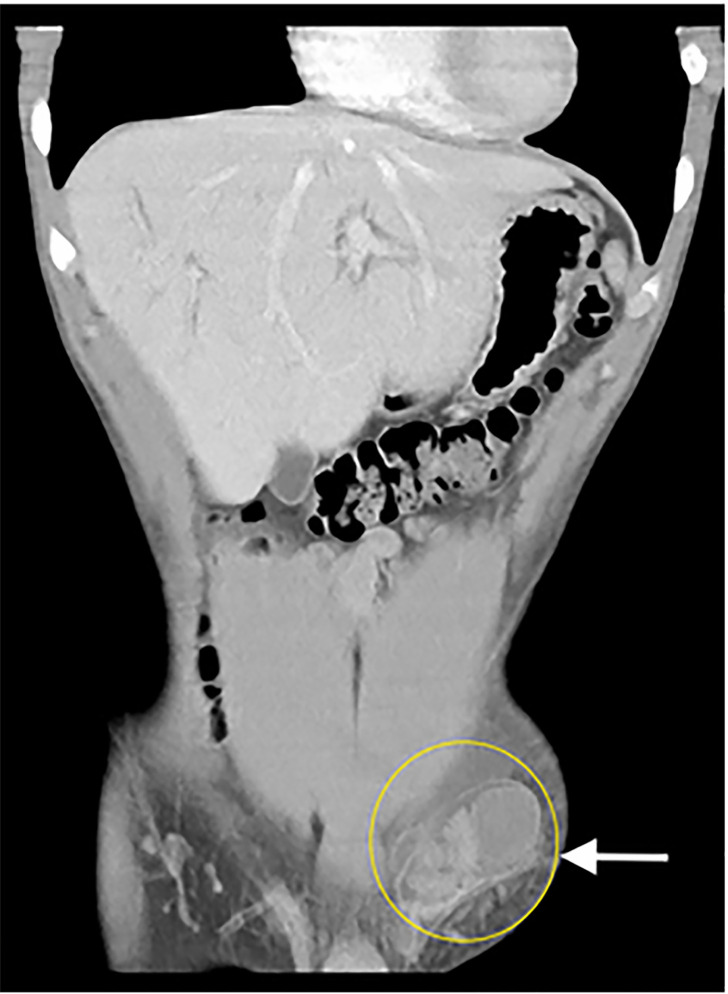
Coronal reconstruction computed tomography demonstrating the cryptorchid testicle in the left inguinal canal (circle).

Laboratory studies including urinalysis were unremarkable. Computed tomography of the abdomen and pelvis was obtained, which revealed an undescended testicle in the left inguinal canal with adjacent edema, as seen in Images 1 and 2.

**Image 3:**
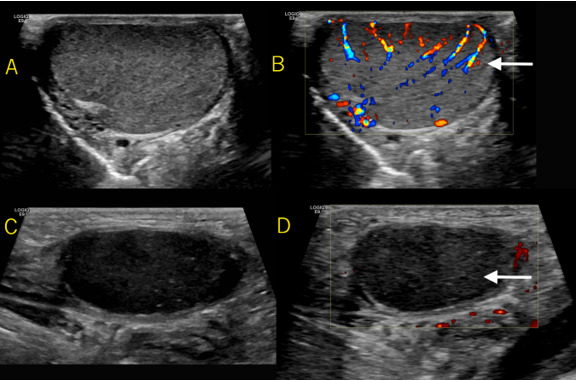
Frame A shows the normal right testicle with the diffuse internal color Doppler signal present seen in Frame B (see arrow). Frame C shows a hypoechoic testicle in the left inguinal canal with absent internal power Doppler signal demonstrated in Frame D (see arrow).

The patient was reassessed, and testicular exam was performed revealing a normal right testis and empty left hemiscrotum. At this point, the patient and his mother confirmed history of undescended left testicle. Testicular Doppler ultrasound revealed a hypoechoic testicle with absence of blood in the left inguinal canal, as seen in Image 3.

Urology was consulted and promptly took the patient to the operating room. The left undescended testis was found to be nonviable with 720 degrees of torsion of the spermatic cord. A left orchiectomy and prophylactic right orchiopexy were performed.

## DISCUSSION

Testicular torsion is a urologic emergency with annual incidence less than 0.004% for males **≤**18.^2^ Torsion must be promptly identified and rapidly treated due to risk of ischemia and infertility. Ideally, surgical management should be completed within six hours as testicular salvage rates are reportedly 90% or greater within this window.^3^,^4^ Beyond six hours, salvage rates progressively decline and are virtually zero by 48 hours.^5^

Cryptorchidism, or undescended testis, affects 2–4% of full-term males with higher incidence seen in preterm infants.^6^ Orchiopexy is ideally performed within the first year of life due to increased risk of infertility and malignancy.^6^,^7^ Interestingly, the first described case of testicular torsion was in 1840 by Delasiauve in a 15-year-old male with cryptorchid testis.^8^ Overall, torsion of an undescended testis is rare, most commonly occurring in pediatric patients, particularly during the perinatal period.^7^ The exact risk of torsion with cryptorchid testis is still unknown, although some articles suggest it may be up to 10 times more likely than torsion of a descended testis.^8^,^9^

**CPC-EM Capsule**

What do we already know about this clinical entity?

*Testicular torsion is a relatively uncommon phenomenon that typically presents with scrotal pain. Torsion must be promptly identified and surgically managed within six hours*.

What makes this presentation of disease reportable?

*Torsion of a cryptorchid testis is exceedingly rare and presents differently than torsion of descended testis, often with lower abdominal pain*.

What is the major learning point?

*A high index of suspicion is necessary to make this diagnosis, as the etiology of undifferentiated lower abdominal pain is vast*.

How might this improve emergency medicine practice?

*A thorough genitourinary exam in patients presenting with lower abdominal pain will aid in diagnosis and improve patient care*.

The clinical presentation of undescended testicular torsion includes lower abdominal pain, vomiting, and decreased oral intake. Physical examination typically reveals inguinal swelling with a firm, tender mass and empty ipsilateral hemiscrotum.^7^,^8^ Doppler ultrasonography, computed tomography, and technetium Tc-99m scrotal scintigraphy can aid in diagnosis.^7^ Torsion of cryptorchid testicle is more commonly left sided.^9^ According to case review, average time from symptom onset to hospital evaluation was 48 hours.^7^ Subsequently, rates of salvage are substantially lower at 10% in cryptorchid torsion.^9^

## CONCLUSION

Torsion of a cryptorchid testicle is an uncommon phenomenon that clinicians should be aware of and must include in their differential for abdominal pain. Torsion is one of the few urologic emergencies. A high index of suspicion is required to make this diagnosis as it may imitate other acute abdominal emergencies including incarcerated inguinal hernia. Lower abdominal pain should always prompt consideration of genitourinary pathology. A thorough physical examination of the genitourinary tract should decrease diagnostic error and improve patient care in such cases.
